# VOC fingerprints: metabolomic signatures of biothreat agents with and without antibiotic resistance

**DOI:** 10.1038/s41598-020-68622-x

**Published:** 2020-07-16

**Authors:** Allyson Dailey, Jessica Saha, Fatima Zaidi, Hafsa Abdirahman, Amanda Haymond, Farhang Alem, Ramin Hakami, Robin Couch

**Affiliations:** 1 Department of Chemistry and Biochemistry, George Mason University, Manassas, VA USA; 2 National Center for Biodefense and Infectious Diseases, School of Systems Biology, George Mason University, Manassas, VA USA

**Keywords:** Metabolomics, Metabolomics

## Abstract

Category A and B biothreat agents are deemed to be of great concern by the US Centers for Disease Control and Prevention (CDC) and include the bacteria *Francisella tularensis*, *Yersinia pestis*, *Burkholderia mallei*, and *Brucella* species. Underscored by the impact of the 2020 SARS-CoV-2 outbreak, 2016 Zika pandemic, 2014 Ebola outbreak, 2001 anthrax letter attacks, and 1984 Rajneeshee *Salmonella* attacks, the threat of future epidemics/pandemics and/or terrorist/criminal use of pathogenic organisms warrants continued exploration and development of both classic and alternative methods of detecting biothreat agents. Volatile organic compounds (VOCs) comprise a large and highly diverse group of carbon-based molecules, generally related by their volatility at ambient temperature. Recently, the diagnostic potential of VOCs has been realized, as correlations between the microbial VOC metabolome and specific bacterial pathogens have been identified. Herein, we describe the use of microbial VOC profiles as fingerprints for the identification of biothreat-relevant microbes, and for differentiating between a kanamycin susceptible and resistant strain. Additionally, we demonstrate microbial VOC profiling using a rapid-throughput VOC metabolomics method we refer to as ‘simultaneous multifiber headspace solid-phase microextraction’ (simulti-hSPME). Finally, through VOC analysis, we illustrate a rapid non-invasive approach to the diagnosis of BALB/c mice infected with either *F. tularensis* SCHU S4 or *Y. pestis* CO92.

## Introduction

Category A and B biothreat agents include several bacteria, viruses, and toxins deemed to be of great concern by the US Centers for Disease Control and Prevention (CDC), due to their ease of dissemination/transmission, morbidity/mortality rates, and ability to cause public panic and disruption. Underscored by the impact of the 2020 SARS-CoV-2 outbreak, the 2016 Zika pandemic, the 2014 Ebola outbreak, the 2001 anthrax letter attacks, and the 1984 Rajneeshee *Salmonella* attacks, the threat of future epidemics/pandemics and/or terrorist use of pathogenic organisms warrants continued exploration and development of both classic and alternative methods of rapidly detecting agents of threat.

Although sensitive and selective detection techniques such as polymerase chain reaction (PCR), microbial dichotomous keying, and/or enzyme-linked immunosorbent assays (ELISA) are well established^1^, these techniques are typically time consuming, laborious, and costly. Conversely, bacterial metabolome fingerprints, such as those derived through the use of techniques like Matrix-Assisted Laser Desorption Ionization (MALDI) coupled with mass spectrometry (MS), are rapid, reproducible, and cost effective^2,3^. Using gas chromatography coupled with mass spectrometry (GC–MS), we have previously demonstrated the diagnostic potential of volatile organic compounds (VOCs) emanating from biological samples^4–7^. VOCs comprise a large and highly diverse group of carbon-based molecules, generally related by their volatility at ambient temperature. A VOC-based diagnostic approach is an attractive method of detecting biothreat agents, as it holds promise for rapid non-invasive diagnostics and real-time monitoring. Encouragingly, studies comparing VOCs produced by sepsis-inducing bacteria have identified metabolite biomarkers distinguishing *Staphylococcus aureus, Pseudomonas aeruginosa,* and *Escherichia coli*^8–10^. Further, differentiation between strains of *S. aureus* and mycobacteria have also been achieved through VOC profiling^11,12^. VOC profiling has also been shown to be a valuable tool in the differentiation of antibiotic resistant strains of *Klebsiella pneumoniae* and *Enterobacter cloacae*^13^.

In light of their eminence as Category A/B biothreat agents, we sought here to assess if microbial VOC (mVOC) fingerprints, generated by GC-based global (untargeted) metabolomic profiling, can uniquely differentiate liquid cultures of *Francisella tularensis* (the causative agent of tularemia), *Burkholderia pseudomallei* (melioidosis), *Brucella melitensis* (brucellosis), and *Yersinia pestis* (plague). The metabolomic profiling was accomplished with multifiber headspace solid phase microextraction (multi-hSPME), in combination with a gas chromatograph furnished with a flame ionization detector (GC-FID). As detailed within, the resulting mVOC fingerprints were found to clearly distinguish these bacteria. Hence, we then sought to test the influence of select environmental effectors on the culture-derived mVOC fingerprints, as well as to shorten the duration of analysis by fabricating an extraction device permitting simultaneous multifiber hSPME (i.e. simulti-hSPME). Accordingly, we used simulti-hSPME, in conjunction with both GC-FID and GC–MS, to derive mVOC fingerprints that differentiate between shake flask cultures of bacteria, including antibiotic sensitive and antibiotic resistant strains of *F. tularensis* and *Y. pestis*. However, using an animal model of infection, simulti-hSPME failed to distinguish healthy mice from those that were infected with either *F. tularensis* or *Y. pestis*. Alternatively, thermal desorption (TD) coupled with GC–MS enabled us to derive mouse-associated VOC profiles that differentiate the healthy mice from infected mice, and mice infected with an antibiotic sensitive strain of *Y. pestis* from those infected with an antibiotic resistant strain.

## Materials and methods

### Bacterial strains, culture media, and culture conditions

The following reagents were obtained through the NIH Biodefense and Emerging Infections Research Resources Repository, NIAID, NIH: *Francisella tularensis spp. novicida*, Strain U112, NR-13; *Francisella tularensis spp*. *tularensis*, Strain NIH B38, NR-50; *Yersinia pestis*, Strain A1122; *Yersinia pestis,* Strain CO92; *Burkholderia cenocepacia*, Strain LMG 16656, NR-701; and *Brucella neotomae*, Strain 5K33, NR-684. The bacteria were cultured using Tryptic Soy Broth (TSB) supplemented with 0.1% cysteine (TSBC), modified Muller-Hinton media (mMH; 0.025% ferric pyrophosphate, 1 mM CaCl_2_, 1 mM MgCl_2_, 0.1% glucose, and 2% Isovitalex supplement), Heart Infusion Broth (HIB) supplemented with 2.5 mM CaCl_2_ and 2% DL-Xylose, or a defined minimal media (Modified Chamberlin’s Defined Media (MCDM) containing 0.4 g/L L-Arginine, 0.4 g/L L-Aspartic Acid, 0.2 g/L L-Cysteine, 0.2 g/L L-Histidine, 0.4 g/L L-Isoleucine, 0.4 g/L L-Leucine, 0.4 g/L L-Lysine, 0.4 g/L L-Methionine, 2.0 g/L L-Proline, 0.4 g/L L-Serine, 2.0 g/L L-Threonine, 0.4 g/L L-Tyrosine, 0.4 g/L L-Valine, 0.04 g/L Spermine disphosphate, 0.004 g/L Thiamine HCl, 0.002 g/L L-Calcium pantothenate, 4.0 g/L Glucose, 10 g/L NaCl, 0.135 g/L MgSO_4_·7H_2_O, 1.0 g/L KH_2_PO_4_, 1.0 g/L K_2_HPO_4_, 1.92 g/L Sodium Citrate, 0.002 g/L FeSO_4_·7H_2_O, and pH adjusted to 6.2). Agar was added (20 g/L) to prepare solid media. All work was conducted using aseptic techniques, following approved protocols.

Individual bacterial colonies isolated from TSBC, sheep blood, or mMH agar plates were used to inoculate corresponding liquid cultures. The bacteria were typically cultured in 10 mL of media in loosely capped 50 mL Falcon conical tubes incubated for 18 h at 37 °C, 250 rpm, but they also culture well in 2 mL media in foam capped 15 mL Falcon conical tubes incubated for 8 h at 37 °C, 250 rpm. Fully virulent *Y. pestis* Strain CO92 was cultured in 10 mL of media in a fully capped parafilm sealed 50 mL Falcon conical tube and incubated for 18 h at 28 °C, 250 rpm. The 18 h cultures served as seed cultures, from which an inoculum was prepared by adjusting the optical density at 600 nm (OD_600_) of the culture to 1.0 with fresh media. Then, a 500 µL aliquot of the inoculum was added to 25 mL of fresh media contained in a capped 125 mL flask. The flask was incubated under the same conditions for 24 h then aliquots (250 µL) were aseptically removed, dispensed into amber vials (VWR Screw Top Vial Amber Glass 15 × 45 mm; 4 mL), and snap frozen in liquid nitrogen. The vials were stored at − 80 °C until analyzed.

When evaluating select media alterations, cells from an overnight TSBC culture were harvested by centrifugation (4 °C, 3,800×*g*, 15 min), washed three times with 1 mL of MCDM (cells were collected by centrifugation after each wash), then resuspended to an OD_600_ = 1.0 in MCDM or MCDM supplemented with either 25 mM MgCl_2_, 12.5 mM NiCl_2_, or 6.25 mM NaCl. Cultures were incubated in a foam capped 125 mL Erlenmeyer flask at 37 °C, 250 rpm, for 8 h, then dispensed in 250 μL aliquots into amber vials, snap frozen in liquid nitrogen, and stored at − 80 °C until analyzed.

Kanamycin resistant strains were acquired via electroporation. The following reagent was obtained through the NIH Biodefense and Emerging Infections Research Resources Repository, NIAID, NIH: Shuttle Vector pFNLTP1 for Gene Expression in *Francisella* Species and *Escherichia coli*, NR-4194. The plasmid pHSG298 was obtained from Clontech. *Y. pestis* with pHSG298 was cultured using TSBC supplemented with 50 µg/mL kanamycin. *F. tularensis* with pFLNTP1 was cultured in mMH with the addition of 10 µg/mL of kanamycin.

*Yersinia pestis* competent cells were prepared as previously described, but with a few modifications^14^. Overnight liquid cultures of *Y. pestis* A1122 (TSBC, 28 °C, 250 rpm) or *Y. pestis* CO92 (HIB, 28 °C, 250 rpm) were diluted 1:50 in 25 mL of fresh media and allowed to incubate at 28 °C, 250 rpm to an OD_600_ of 0.5 (approximately 3 h). Cells were then harvested by centrifugation (4,000×*g* for 10 min at 4 °C), washed once with sterile MilliQ water, then once with sterile transformation buffer (15% glycerol, 272 mM sucrose). Following the second wash, the cells were resuspended in 400 µL of transformation buffer, divided into 40 µL aliquots, snap frozen, and stored at − 80 °C until used.

Transformation of *Y. pestis* was conducted essentially as described previously^14^*.* A 40 µL aliquot of competent cells was combined with 5 µL of pHSG298 (~ 100 ng/µL) and placed on ice for 1 min. The mixture was then transferred to an ice-cold 0.2 cm electroporation cuvette. Cells were electroporated with a single electric pulse (25 µF, 200 Ω) at a field strength of 12.5 kV/cm. Transformed cells were then added to 1 mL of SOC media and incubated at 28 °C, 250 rpm for 2 h. Transformants were identified by plating on TSBC agar plates with 50 µg/mL kanamycin; transformant colonies were visible within 24 h.

*Francisella tularensis* competent cells were prepared essentially as previously described, but with some modification^15^. *F*. *tularensis* NIH B38 was cultured in mMH at 28 °C, 200 rpm for 3 days. The culture was then diluted 1:10 in 80 mL fresh mMH and allowed to incubate until an OD_600_ of 0.6 was obtained (approximately 3 days). Cells were harvested by centrifugation (4,000×*g* for 10 min at 4 °C), then washed twice with sterile transformation buffer (500 mM sucrose). Cells were resuspended in 400 µL of transformation buffer and were used the same day for transformation.

Transformation of *F. tularensis* was conducted as described previously, with some modification^15^. A 200 µL aliquot of competent cells was combined with 2 µL of pFNLTP1 (~ 100 ng/µL) then incubated at room temperature for 10 min. The mixture was transferred to an ice-cold 0.2 cm electroporation cuvette and the cells were electroporated with a single electric pulse (25 µF, 600 Ω) at a field strength of 12.5 kV/cm. Transformed cells were added to 1 mL of mMH broth and incubated at 37 °C, 250 rpm for 6 h. Transformants were identified by plating on mMH agar plates with 10 µg/mL kanamycin; transformant colonies were visible within 4 days.

The kanamycin sensitive and resistant strains were analyzed using individual bacterial colonies from appropriate agar plates to inoculate a 50 mL Falcon tube containing 10 mL of media (TSBC for the kanamycin sensitive *Y. pestis* strain, TSBC + 50 µg/mL kanamycin for the kanamycin resistant *Y. pestis* strain, mMH for the kanamycin sensitive *F. tularensis* strain, and mMH + 10 µg/mL kanamycin for the kanamycin resistant *F. tularensis* strain). Each culture tube was loosely capped and incubated for 18 h at 37 °C, 250 rpm. An inoculum culture was then prepared using fresh sterile media to adjust the OD_600_ of each to 1.0. A 500 µL aliquot of the inoculum culture was then added to 25 mL of appropriate media for the experiment (TSBC or mMH, with or without kanamycin) contained in a loosely capped 125 mL flask. The flasks were incubated for 24 h at 37 °C, 250 rpm. Aliquots (250 µL) were aseptically removed from the flasks and snap frozen in amber vials for subsequent analysis. Alternatively, the analysis was performed with our simulti-hSPME device placed on the mouth of the culture flask, permitting extraction of the mVOCs directly from within the flask.

### hSPME derived mVOC fingerprints

Developed by the Pawliszyn group^16,17^, the hSPME process involves exposing a sorbent coated fiber to the headspace above a sample to facilitate the extraction and concentration of VOCs. The volatile analytes emanating from the sample enter the headspace where they interact and associate with the polymeric coating on the SPME fiber. To perform hSPME, sample vials containing frozen culture aliquots were preheated to 37 °C for 30 min. Next, a hSPME fiber assembly was manually positioned into the headspace through the septum cap above the culture and the fiber exposed to the volatiles for 60 min (the sample vial temperature was held at 37 °C for the duration of the exposure). The fiber assembly was then placed into the GC inlet for thermal desorption of the analytes. The following hSPME fibers (Supelco, Bellefonte, PA) were used in the investigation; CAR/PDMS 85 µm with Stableflex, 100 µm PDMS, PDMS/DVB 65 µm with Stableflex, PA 85 µm, DVB/CAR/PDMS 50/30 µm, PEG 60 µm. All fibers were preconditioned before use, per the manufacturer’s instructions. All analyses were performed in triplicate.

The environmental effector investigation was conducted using the same procedure with the following hSPME fibers (Supelco, Bellefonte, PA): PDMS/DVB 65 μm, PA 85 μm, CAR/PDMS 75 μm, CAR/PDMS 85 μm with Stableflex, PDMS 100 μm, PDMS 7 μm, PEG 60 μm, and DVB/CAR/PDMS 50/30 μm with Stableflex.

The hSPME-based comparison of the kanamycin sensitive and resistant bacteria was performed as described above, but with the following hSPME fibers (Supelco, Bellefonte, PA); CAR/PDMS 85 µm with Stableflex, 100 µm PDMS, PDMS/DVB 65 µm with Stableflex, PA 85 µm, DVB/CAR/PDMS 50/30 µm, PEG 60 µm. The analysis was also performed with these fibers in conjunction with our simulti-hSPME device, placed on the mouth of the culture flask to permit analysis of the mVOCs directly within the flask.

### TD derived mVOC fingerprints

Colonies were removed from the plate and a suspension was made using liquid HIB media. The OD_600_ was adjusted with sterile media to 1.2. 660 μL aliquots of the bacterial suspension were used to inoculate three 125 mL plastic baffled Corning flasks, each containing 33 mL of liquid media. The flasks were placed onto the shaker platform and shaken at 100 rpm, 28 °C overnight. The following morning, the OD_600_ was read and the flasks were placed on a 37 °C hot plate for 5 min. Next, the VOCs from the culture were pulled from the culture and into an airtight 10 L ALTEF gas sampling bag (Restek). Following the collection of the VOCs, the sample in the bag was purged into a previously preconditioned 100 mg Tenax TA Thermal Desorption tube. Following the collection into the tube, the tube was brought to the GC–MS and analyzed.

### Culture conditions for aerosol challenge

Samples were prepared as previously described^18^ with some modifications. Colonies were removed from the plate and a suspension was made using liquid HIB media. The OD_600_ was adjusted with sterile media to 1.2. 660 μL aliquots of the bacterial suspension were used to inoculate six 125 mL plastic baffled Corning flasks, each containing 33 mL of liquid media. A fourth flask with 33 mL of liquid media served as a sterility control and was not inoculated. The flasks were placed onto the shaker platform and shaken at 100 rpm, 28 °C for 24 h. The following day, the flasks with the cultures were aseptically transferred into 50 mL Falcon tubes and placed inside individually sealed secondary containers. The secondary containers were placed into a Thermo Scientific 8 × 50 mL individually sealed fixed angle rotor seated within a Sorval ST-16 centrifuge and centrifuged for 10 min at 5,000 rpm. Following centrifugation, the supernatant was removed and the pelleted cells were resuspended in a total volume of 10 mL of fresh sterile HIB media. 30 μL of antifoam solution was added to the culture and a second tube of 10 mL of fresh sterile HIB media, which was used as the media blank. Two additional tubes of fresh sterile HIB media with 30 μL of antifoam solution were used for the all glass impinger (AGI).

### Aerosol challenge of the mice

All infections were conducted using an animal protocol approved by the George Mason University Institutional Animal Care and Use Committee (Protocol Number: 379). Female 6 week old BALB/c mice (Envigo) were exposed to either the pathogen (*Y. pestis* CO92 or *F. tularensis* SCHU S4) or the HIB media blank in a whole body aerosol chamber and exposure system (Biaera Technologies, Frederick, MD) within a class III hood line. Using a 3-jet collision nebulizer, the mice were exposed to the pathogen for 30 min at a chamber flow rate of 19.5 lpm. The resulting aerosolized material was collected in an all glass impinger (AGI) and plated following the exposure.

### TD derived whole body VOC fingerprints

24 h after exposure (day 1 post infection) and every 24 h thereafter, the whole body VOCs were collected into 10 L ALTEF gas sampling bags (Restek). Specifically, on the inflow of a vacuum pump, a tube with a disposable pipette tip was connected to a disposable HEPA filter. On the outflow of the same pump, the gas sampling bag was connected. The mice cages were brought one at a time into the biohood and the whole body VOCs were collected into the gas sampling bag. Following the collection, the cages were returned to the racks. The contents of the bag were then purged into a previously preconditioned 100 mg Tenax TA (Sigma) Thermal Desorption tube (SIS, Ringoes, NJ). The Thermal Desorption tube was then sealed with airtight stainless steel caps and analyzed.

### Instruments

Samples were analyzed using an Agilent 6890 Plus GC-FID equipped with an Agilent DB5-MS capillary column (15 m in length, 0.25 mm ID, and 0.25 μm film thickness) or an Agilent 6890 Plus GC-FID equipped with a Restek RXI-5Sil MS capillary column (30 m in length, 0.25 mm ID, and 0.5 μm film thickness). Each instrument was fit with a 0.75 mm ID SPME injection port liner and was operated in splitless mode at varying inlet temperatures (Table 1). Helium carrier gas was used at a flow rate of 1.5 mL/min. The GC oven was held at an initial temperature of 35 °C for 1 min, ramped to 80 °C at 3 °C/min, then to 120 °C at 10 °C/min, next to 260 °C at 40 °C/min and held for 2 min, and finally to 280 °C at 40 °C/min. The final temperature of 280 °C was held for 2.5 min. The total run time for the analysis was 30 min. Shorter run times were achieved using an initial GC oven temperature of 35 °C for 1 min, ramped to 50 °C at 3 °C/min, then to 300 °C at 27.5 °C/min (the total run time for this analysis is 15.09 min).

**Table 1:** SPME fiber operational conditions.

Fiber	Hub color	Inlet temperature (°C)	Precondition time (min)
CAR/PDMS 75 μm	Black	300	60
PDMS/DVB 65 μm	Blue	250	30
PDMS 7 μm	Green	320	60
DVB/CAR/PDMS 50/30 μm Stableflex	Grey	270	60
CAR/PDMS 85 μm Stableflex	Light blue	300	60
PDMS/DVB 65 μm Stableflex	Pink	250	30
CW (PEG) 60 μm	Purple	240	30
PDMS 100 μm	Red	250	30
PA 85 μm	White	280	60

The GC–MS used in this investigation was an Agilent 5977B MSD equipped with an Agilent HP-5MS ultra inert column, 30 m in length, 0.25 mm ID, and 0.25 μm film thickness, and an ultra-inert SPME injection port liner operated in splitless mode at varying inlet temperatures (Table 1). Helium carrier gas and GC oven conditions were identical to those described above, with a total run time of 30 min and 15.09 min, respectively.

For the simulti-hSPME analyses, the chromatograms were generated with the GC oven held at an initial temperature of 30 °C for the initial desorption period. Each fiber was sequentially desorbed for 1 min at their manufacturer-recommended temperature (Table 1). Following desorption of the final fiber, the oven temperature was increased at 15 °C/min to a maximum temperature of 325 °C, at which it was held for another 2 min. The total run time was 23.67 min.

For the Thermal Desorption analyses, the GC–MS used in this investigation was an Agilent 5977B MSD equipped with an Agilent HP-5MS ultra inert column, 30 m in length, 0.25 mm ID, and 0.25 μm film thickness, and an Agilent 935 μL focus liner with deactivated glass wool operated in split mode (0.1:1 split ratio). The VOCs were desorbed from the thermal desorption tube using the SIS short path thermal desorption system at an inlet temperature of 300 °C for 15 min. Using a cryotrap set at − 60 °C, desorbed analytes were cryogenically held at the head of the column. Following desorption, the cryotrap was heated to 300 °C to release the volatile analytes. The chromatograms were generated with the GC oven temperature held at an initial temperature of 35 °C for the initial desorption period and 1 min thereafter. The oven temperature was then increased at a rate of 10 °C/min to a maximum temperature of 300 °C, at which it was held for another 2 min. The total run time was 29.5 min.

### Data analysis

Chromatograms were converted into binary plots via SciLab utilizing a custom script written in the MatLab technical computing language. To expedite the throughput of binary plot generation, we wrote a custom Perl script to automatically generate the binary plots. Following a sample run, the peaks in the chromatogram were integrated using Agilent Technologies’ ChemStation software (version Rev. A.09.01; for GC-FID) or Agilent Technologies’ MassHunter Qualitative Analysis software (version B.07.00; for GC–MS). The information was converted into a binary matrix (where 1 denotes the presence of a peak and 0 an absence at the specific retention time). The resulting matrix was plotted using white and black horizontal bars to denote a peak presence or absence, respectively. To compile our replicate runs into one composite binary plot, we imposed a frequency filter for peak inclusion in the binary plot (e.g. for triplicate runs the peak needed to appear in at least 2 of the 3 samples to be included in the binary plot). In addition, the binary plots only contain analytes attributed to the bacteria, as signals from the media alone are subtracted (i.e. the binary plots are blank subtracted). Finally, PCA plots, generated using the R statistical software, were used to aid in the differentiation of mVOCs.

To further analyze GC–MS data, features were identified from the chromatogram using the National Institute of Standards and Technology (NIST, Washington, DC) Automated Mass Spectral Deconvolution and Identification System (AMDIS) software and mass spectral library (NIST17). As previously described^4,5,7^, only those features with an 85% or greater match to a compound in the spectral library were included in the analysis. Data was consolidated into a master spreadsheet consisting of sample names, feature names, their corresponding intensities, retention times, and weighted AMDIS score. Data was frequency filtered (appearance of 2/3 for bacteria cohort studies; 2/2 for time cohorts in animal infection studies), missing values were replaced with the median value of the cohort, and outliers were identified in each cohort. In all bacterial samples data was z-score standardized and then a PCA was generated with the results. With the animal infection data, the data was total TIC normalized, log transformed, then pareto scaled. The resulting pareto scaled data was used to generate a PCA.

## Results and discussion

### mVOC differentiation of biothreat agents

**Figure 1:**
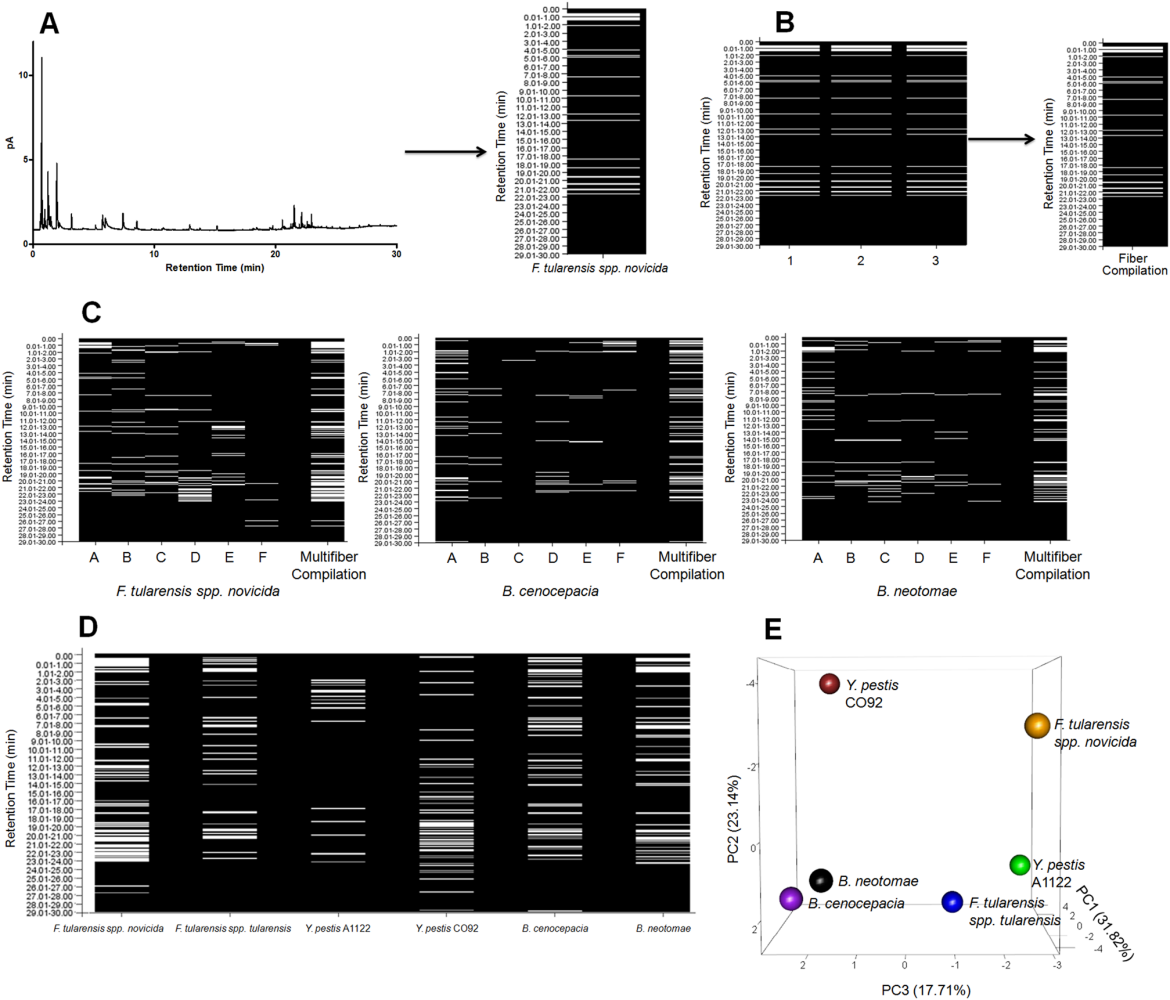
The mVOC fingerprints derived from liquid cultures of select bacteria. (**A**) The conversion of a GC-FID chromatogram into a binary plot. On the left is the chromatogram obtained from a single 60 min DVB/CAR/PDMS 50/30 μm Stableflex fiber hSPME extraction of an aliquot of an *F. tularensis spp. novicida* liquid culture. Using a computer script written in-house, the chromatogram is converted into the binary plot shown to the right of the chromatogram. The binary plot resembles a universal product code (UPC) which uniquely identifies products in retail stores. In the binary plot, the white bars are indicative of a chromatographic peak at a specified retention time (y-axis). The binary plot (i.e. mVOC fingerprint) is blank subtracted by removing VOCs derived from media alone (identified in a parallel extraction performed with the same SPME fiber type and sterile culture media; not shown). (**B**) Combining replicate binary plots for a fiber type. The three binary plots shown reflect three replicate extractions performed using aliquots obtained from three different cultures of *F. tularensis spp. novicida*. Each of the three extractions were completed with a DVB/CAR/PDMS 50/30 μm Stableflex fiber and are each blank subtracted. The replicate extractions (1, 2, and 3) are indicated along the x-axis, while the y-axis indicates the retention time of the chromatographic peaks (minutes). The arrow depicts how the replicates are combined into one fiber compilation binary plot for that fiber type. Note that for this particular analysis, the binary plots derived from each replicate were identical. Replicates derived from other bacterial cultures with other fiber types are typically > 90% identical to one another. Only peaks present in two or more replicate chromatograms are included in the fiber compilation binary plot for that hSPME fiber type. (**C**) A 6-fiber hSPME analysis of mVOCs derived from *F. tularensis spp. novicida*, *B. cenocepacia*, and *B. neotomae* liquid cultures. With each bacterium, the 6 fiber type binary plots (each created from triplicate extractions, as described in panel (**B**) above) are further combined into a multifiber compilation fingerprint, reflective of the overall mVOC metabolome composition. A 60 min extraction duration was used with each of the following fiber types: A—DVB/CAR/PDMS 50/30 μm Stableflex, B—CAR/PDMS 85 μm Stableflex, C—PDMS/DVB 65 μm Stableflex, D—CW (PEG) 60 μm, E—PDMS 100 μm, F—PA 85 μm. (**D**) A comparison of 6-fiber compilation plots derived from liquid cultures of the indicated bacteria. Each compilation plot is media blank subtracted. The hSPME-derived mVOC fingerprints uniquely identify each of the bacteria among the others. (**E**) Principal component analysis (PCA) of the derived mVOC metabolomes readily differentiates the bacteria, as reflected in the PCA plot shown. The mVOC metabolomes analyzed are the same as those derived in D.

To ascertain whether VOCs exuded by the microorganisms *F. tularensis spp. novicida*, *F. tularensis spp. tularensis, Y. pestis* A1122, *Y. pestis* CO92*, B. cenocepacia*, and *B. neotomae* can be used to identify and differentiate these bacteria, aliquots from liquid cultures of each were acquired and then analyzed by multi-hSPME coupled with GC-FID, as detailed in the “Materials and methods” section. Given the anticipated chemical diversity of metabolites within the mVOC metabolome^7^, and to enhance the probability of differentiating the bacterial strains based upon their mVOC fingerprints, we performed a 6-fiber analysis of the mVOCs, using 6 hSPME fibers of differing sorbent composition. The VOCs from each culture aliquot were extracted from the headspace using a single hSPME fiber type, and the resulting GC-FID chromatograms were converted into binary plots to facilitate easy visualization of the derived mVOC profile (Fig. 1A). The extractions performed with each fiber type were done in triplicate, using fresh aliquots from separate cultures for each (Fig. 1B), and only those peaks appearing in 2 or more replicate chromatograms were included in the fiber compilation binary plot for that fiber. Parallel extractions of sterile culture media with each of the fiber types defined the media-associated VOCs (i.e. serve as a blank), which are subtracted from the corresponding bacterial binary plot. A multifiber compilation binary plot is then generated by combining all of the separate fiber compilation binary plots into one plot, as illustrated in Fig. 1C. The result is a comprehensive bacterial mVOC fingerprint, akin to a UPC bar code found on items sold in retail stores.

Figure 1D illustrates the blank subtracted mVOC fingerprints derived from a 6-fiber hSPME GC-FID analysis of *F. tularensis spp. novicida*, *F. tularensis spp. tularensis, Y. pestis* A1122, *Y. pestis* CO92*, B. cenocepacia*, and *B. neotomae* liquid cultures. As seen, the mVOC fingerprints uniquely identify each of the bacterial strains. This differentiation is further emphasized in Fig. 1E, wherein all of the bacterial cultures are clearly segregated within the PCA plot based upon the composition of their mVOC metabolome. Perhaps most noteworthy is the clear separation of *F. tularensis spp. novicida* from *spp. tularensis* and the differentiation between the virulent (CO92) and non-virulent (A1122) strains of *Y. pestis.*

### Influence of environmental effectors on the derived mVOC profile

The mVOC fingerprints reflect microbial metabolism, which in turn reflects environmental conditions, such as media composition. In the same way that altered media composition can hinder microbial identification by MALDI-MS^19,20^, media-induced alteration of the mVOC metabolome may be problematic when using mVOC fingerprints to identify microbes. Environmental effectors such as metal salts are well known to influence the growth and metabolism of microbes, often through the regulation of gene expression^21,22^. Hence, we sought to determine the effect of select metal salts on the derived mVOC fingerprints obtained from representative bacterial cultures.

**Figure 2:**
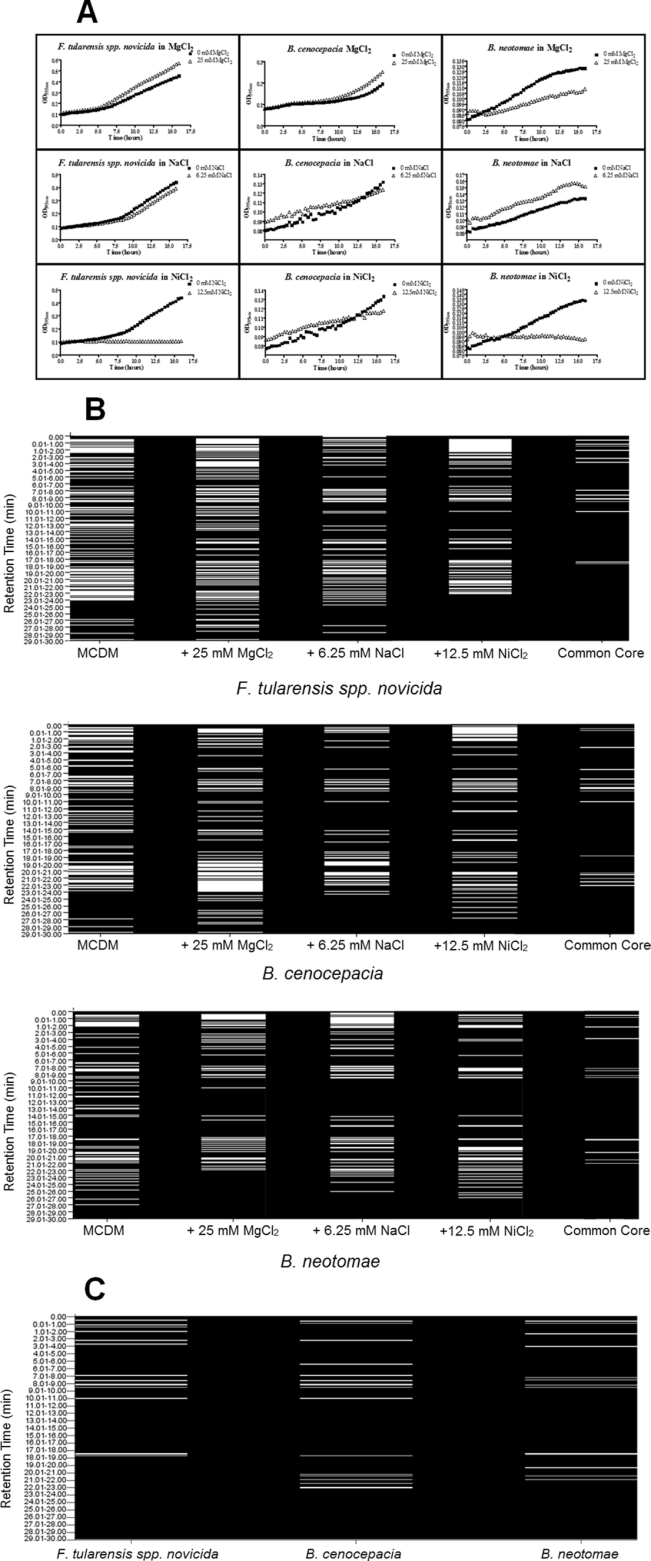
Effectors of bacterial growth and the resulting mVOC fingerprints. (**A**) The effect of added salts on bacterial growth. The indicated bacteria were each cultured in defined liquid media (MCDM) supplemented with various selected effectors [25 mM MgCl_2_, 6.25 mM NaCl, and 12.5 mM NiCl_2_; these concentrations were chosen from a series of growth assays performed with various concentrations of each (data not shown)]. Cultures were incubated at 37 °C and monitored for growth (OD_595_) every 20 min for 16 h. See text for further discussion. (**B**) The corresponding mVOC fingerprints derived from bacterial cultures in defined media (MCDM), defined media supplemented with 25 mM MgCl_2_, defined media supplemented with 6.25 mM NaCl, and defined media supplemented with 12.5 mM NiCl_2_, as indicated. Note that in all tested media compositions, the derived mVOC fingerprint uniquely differentiates the bacteria from one another. The following SPME fibers were used with a 60 min extraction duration: PDMS/DVB 65 μm, PA 85 μm, CAR/PDMS 75 μm, CAR/PDMS 85 μm with Stableflex, PDMS 100 μm, PDMS 7 μm, PEG 60 μm, and DVB/CAR/PDMS 50/30 μm with Stableflex. (**C**) A comparison of the common core mVOC fingerprints illustrates how each of the three bacteria are readily differentiated.

As shown in Fig. 2A, addition of these metal salts to the liquid growth media has varying degrees of influence on the growth of *F. tularensis spp. novicida*, *B. cenocepacia*, and *B. neotomae*. For *F. tularensis spp. novicida*, the addition of either 25 mM MgCl_2_ or 6.25 mM NaCl has a measurable but minimal effect on the cell density of an overnight culture, compared to that of the unaltered media (MCDM) alone. In contrast, the addition of 12.5 mM NiCl_2_ significantly inhibits the growth of *F. tularensis*. For *B. cenocepacia*, the addition of MgCl_2_, NaCl, or NiCl_2_ has a relatively small influence on the overnight growth of the liquid culture. However, for *B. neotomae*, the addition of MgCl_2_ impairs bacterial growth, but not to the magnitude of inhibition demonstrated by NiCl_2._ Further, the addition of NaCl to the *B. neotomae* culture slightly increases the cell density of an overnight culture (possibly by enhancing cell aggregation).

To evaluate the effect of these metal salts on the resulting metabolic profile, mVOCs were extracted from aliquots of the above bacterial liquid cultures and analyzed via multifiber-hSPME coupled with GC-FID. The derived mVOC fingerprints are presented in Fig. 2B. As seen in the Figure, regardless of the media composition, it is noteworthy that the mVOC fingerprints uniquely differentiate the three bacteria from each other. Furthermore, for each bacterium, the addition of the metal salt alters the composition of the resulting mVOC profile, relative to that of their MCDM cultures. For example, when compared to *F. tularensis spp. novicida* cultured in MCDM, the mVOC metabolic fingerprint obtained from the same bacterium in MCDM + 25 mM MgCl_2_, MCDM + 6.25 mM NaCl, and MCDM + 12.5 mM NiCl_2_ are clearly different. This variation in the mVOC fingerprint is also apparent with *B. cenocepacia* and *B. neotomae.* For each bacterium, differential comparison of the mVOC fingerprints obtained in each of the media types permits identification of a core metabolome that is invariant across all of the media types evaluated (identified as “common core” in Fig. 2B). Comparison of these core mVOC fingerprints uniquely differentiates each of the three bacteria from one another (Fig. 2C). Hence, while diverse environmental conditions lead to alterations in bacterial metabolism, it is conceivable that a uniquely identifying core mVOC metabolome might serve as a fingerprint that enables bacterial identification, regardless of media composition. Alternatively, as proposed for MALDI-MS-based identification of microbes^20^, it may be prudent to generate identification databases with mVOC profiles derived from various culture media and conditions. Finally, while the growth of *F. tularensis spp. novicida* and *B. neotomae* is significantly inhibited by the addition of NiCl_2_ (Fig. 2A), it is also noteworthy that a mVOC fingerprint is still obtained, despite the relatively low cell density in the culture.

### Differentiation of wildtype and antibiotic resistant bacteria

**Figure 3:**
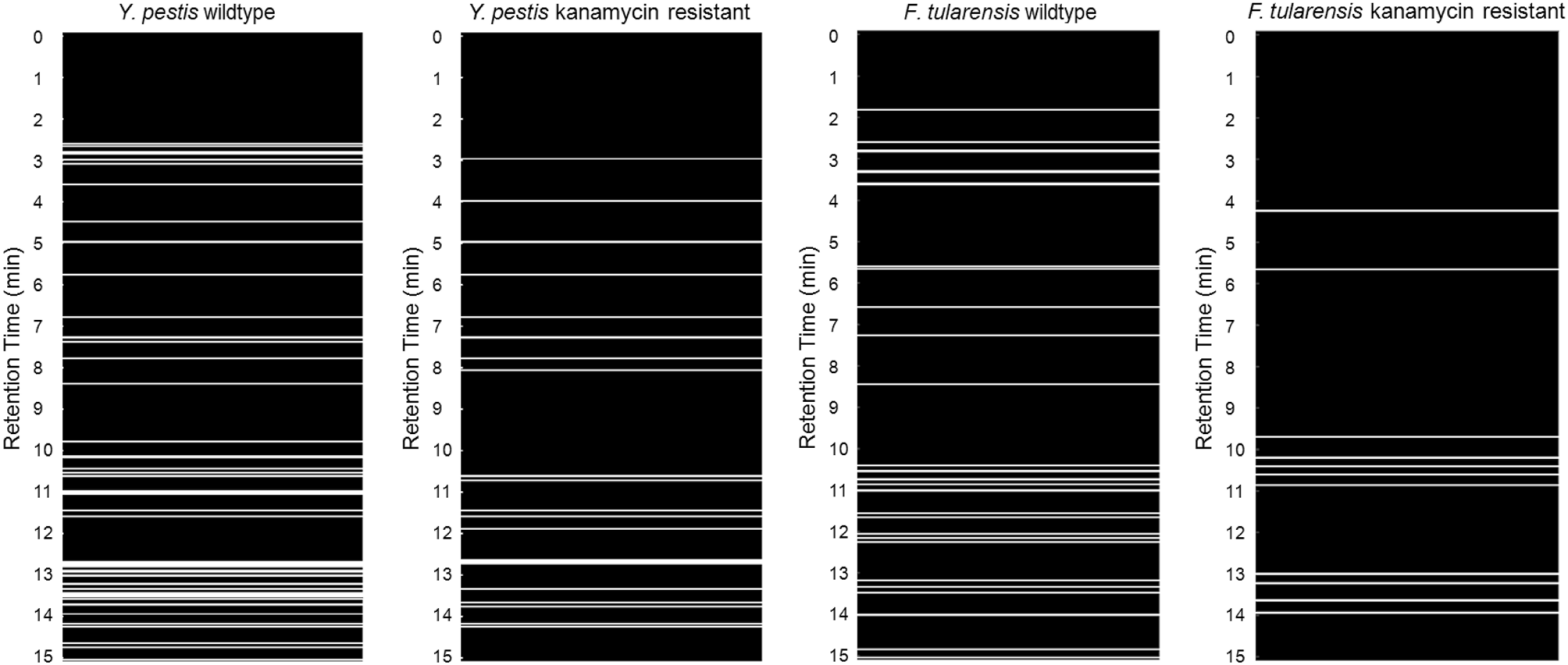
Using the derived mVOC metabolome to differentiate wildtype and kanamycin resistant strains of *Y. pestis* and *F. tularensis*. For each bacterial strain, a 6-fiber analysis was performed with a GC-FID, as described in Fig. 1C, but with a 15 min extraction duration. Based on the derived mVOC fingerprints, the kanamycin resistant strains of *Y. pestis* and *F. tularensis* are readily differentiated from their wildtype counterparts.

In light of the ability of the mVOC fingerprints to differentiate the bacteria, particularly at the subspecies level, we next sought to determine if multifiber hSPME analysis can distinguish kanamycin sensitive (wildtype) from engineered kanamycin resistant strains of *Y. pestis* and *F. tularensis*. Liquid cultures of each were prepared as detailed in the “Materials and methods” section, culture aliquots were collected, and mVOCs extracted via 6-fiber hSPME. The extracted analytes were analyzed via GC-FID, and the resulting chromatograms converted into the multifiber compilation binary plots shown in Fig. 3. As seen in the Figure, the derived mVOC fingerprints clearly differentiate the wildtype and kanamycin resistant strains. While expression of the kanamycin kinase gene (*kan*^*R*^) presumably has an impact on the ATP pool within the bacterial cells, further work is needed to detail exactly how the expression and activity of kanamycin kinase leads to the observed mVOC differences. However, as we elaborate below, an analysis by GC–MS indicates that multiple mVOCs are impacted by *kan*^*R*^ expression.

### Increasing the throughput of mVOC analysis

**Figure 4:**
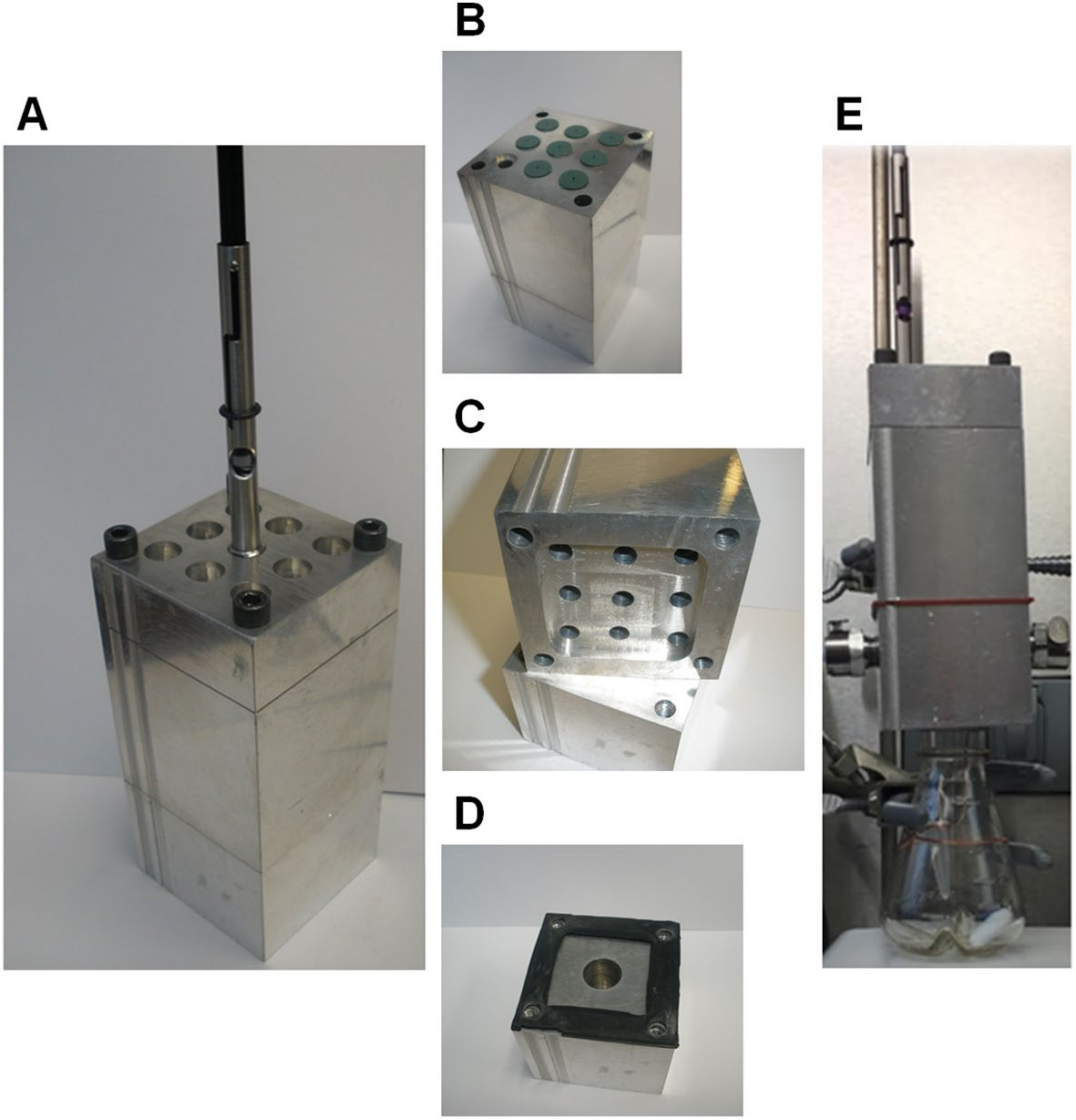
The simulti-hSPME device. (**A**) The device consists of three sections (an upper, middle, and bottom section, held together by 4 bolts). For clarity, the device is shown with only one hSPME syringe in place, but it can accommodate up to nine syringes simultaneously. (**B**) To maintain an airtight seal, each channel/port in the device contains a septum located at the top side of the middle section, through which a hSPME syringe needle can pass (akin to an inlet on a GC). (**C**) A recess has been engineered into the bottom side of the middle section, serving as a headspace above the sample, in which each hSPME sorbent resides when extended and exposed from the syringe needle. (**D**, **E**) A gasket at the top of the bottom section maintains an airtight seal, and a well/port (depending on the configuration) in the bottom section permits mVOCs to pass from a vial/flask directly into the headspace region in the device. The setup for direct sampling of mVOCs from a culture flask is shown in (**E**), with a stir bar and suspended microfuge tube (for an internal standard) seen in the flask.

**Figure 5:**
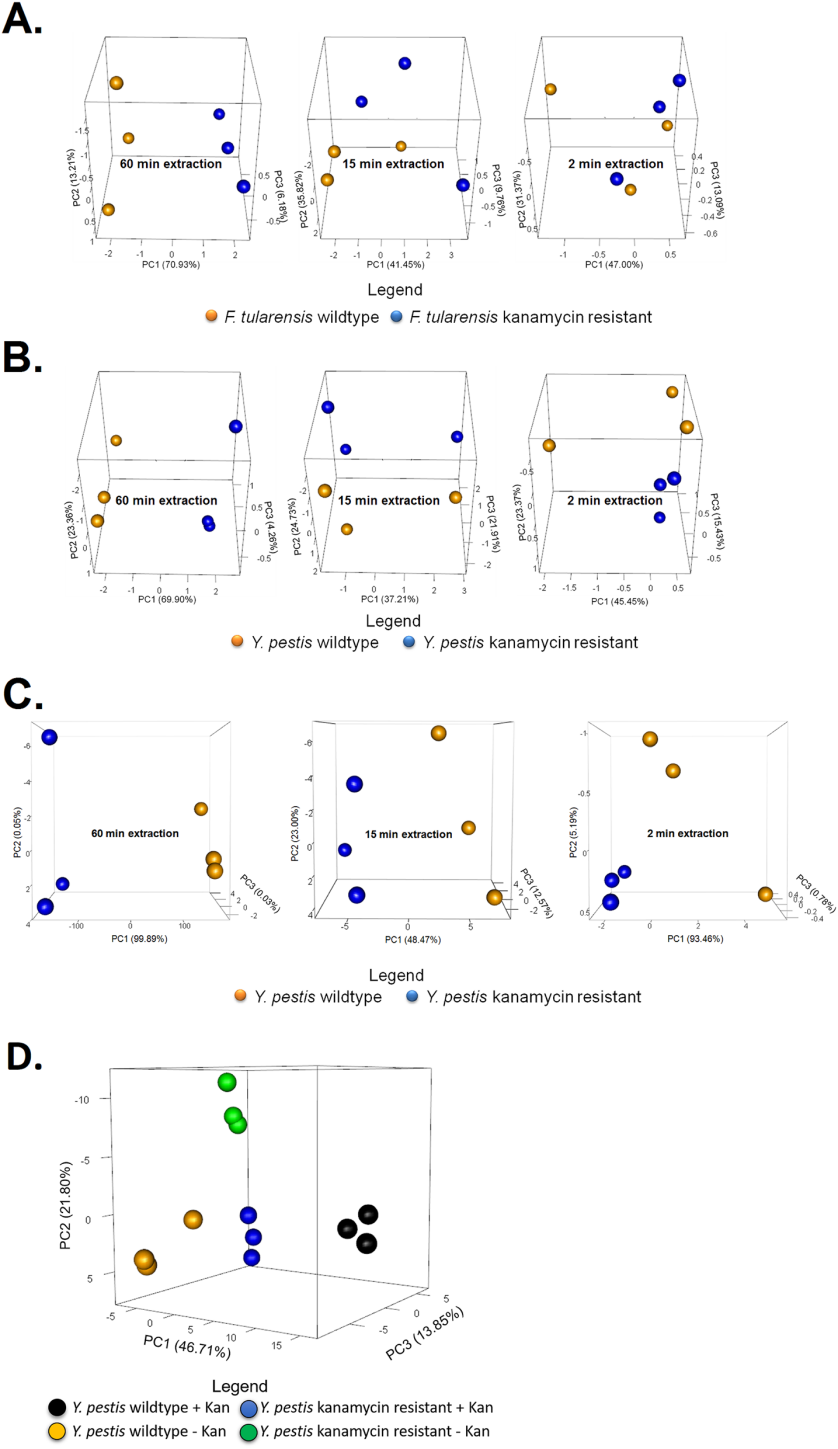
Differentiation and evaluation of extraction duration with wildtype and kanamycin resistant strains of *F. tularensis* and *Y. pestis* using the simulti-hSPME device. Wildtype and kanamycin resistant strains of *F. tularensis* and *Y. pestis* were examined via a 6 fiber simulti-hSPME analysis utilizing either a GC-FID (**A**, **B**) or GC–MS (**C**, **D**). With each sample, the mVOCs were extracted for the indicated extraction duration [60 min, 15 min, or 2 min; 15 min in (**D**)]. PCA plots were generated using the associated mVOC lists [either retention time markers (GC-FID) or putatively identified features (GC–MS)]. GC–MS features were identified using a scoring cutoff of 85% or greater to a molecule in the NIST GC–MS database. The segregation of the samples along PC1 reflects the greatest variation between the corresponding mVOC metabolomes. The analyses were performed in triplicate, with three separate flasks of each bacterial culture. Each sphere in the PCA plot reflects the corresponding derived mVOC metabolome of a bacterial sample, with wildtype cultured in the absence of kanamycin (*F. tularensis* or *Y. pestis*) colored orange, kanamycin resistant cultured in the presence of kanamycin (*F. tularensis* or *Y. pestis*) colored blue, kanamycin resistant *Y. pestis* cultured in the absence of kanamycin colored green, and wildtype *Y. pestis* cultured in the presence of kanamycin colored black. The PCA plots (**A**–**C**) indicate that a 6-fiber analysis using the simulti-hSPME device effectively differentiates the wildtype and kanamycin resistant strains, particularly with the longer extraction durations. Additionally, the PCA plot in (**D**) shows that wildtype *Y. pestis* is significantly differentiated from kanamycin resistant *Y. pestis*, in the presence and absence of the antibiotic. Furthermore, the antibiotic influences the VOC metabolome composition.

While multifiber-hSPME analysis enables successful differentiation of the bacterial strains examined above, a significant drawback of the approach is the time required to perform a 6-fiber extraction (e.g. a 6-fiber analysis, each with a 15 min extraction duration and 30 min chromatography time, performed in triplicate, will take ~ 13.5 h to complete). Thus, to address this, we designed and fabricated an extraction device that allows the use of all hSPME fibers simultaneously, an approach we refer to as simultaneous multifiber-hSPME (simulti-hSPME; Fig. 4). To evaluate the effectiveness of the simulti-hSPME device, we sought to again use the GC-FID to differentiate wildtype and kanamycin resistant strains of *Y. pestis* and *F. tularensis*, but using a 6-fiber simulti-hSPME, performed in triplicate with 60 min, 15 min, or 2 min extraction durations (using a 30 min chromatography time, the time to complete each triplicate analysis is ~ 4.5 h, 2.5 h, and 1.5 h, respectively). As seen in Fig. 5A, a 60 min extraction duration clearly differentiates liquid cultures of wildtype *F. tularensis* from kanamycin resistant cultures, whereas differentiation is less clear using 15 min and 2 min extractions. A parallel analysis of the *Y. pestis* cultures shows similar results; clear differentiation of the wildtype and kanamycin resistant strains with a 60 min extraction duration, but less distinction with the shorter extraction times (Fig. 5B).

As speculated above, expression of the kanamycin kinase gene (*kan*^*R*^) likely has an impact on overall bacterial metabolism, possibly by perturbing the ATP pool within the cell (ATP is a substrate for kanamycin kinase). Further, the *kan*^*R*^ gene in each of these resistant strains resides on a high copy number plasmid, which itself imposes metabolic demands on the cell, thereby altering the metabolome relative to the wildtype strain^23^. Additionally, intracellular metabolic changes have been identified as a bacterial stress response to antibiotic exposure^24^. Collectively, these metabolic alterations will contribute towards the differing mVOC profiles derived from the wildtype and kanamycin resistant strains.

To assess if the plasmid borne *kan*^*R*^ gene and/or the presence of kanamycin alters the resulting mVOC metabolome, we performed a 6-fiber simulti-hSPME analysis of *Y. pestis* cultures using GC–MS. As detailed in the “Materials and methods” section, the resulting chromatograms were subsequently analyzed and mVOCs identified by comparison to a mass spectral library. As seen in Fig. 5C, the derived metabolomes obtained from the wildtype and kanamycin resistant strains clearly differentiate the cultures, even with a 2 min extraction duration. As seen in Fig. 5D, it is apparent that the wildtype strain of *Y. pestis* is distinguished from the kanamycin resistant strain, in both the presence and absence of kanamycin. Similarly, mVOC metabolomes distinguish the kanamycin resistant strain of *Y. pestis* in media with and without the presence of kanamycin. Hence, the plasmid borne *kan*^*R*^ gene alters the resulting mVOC metabolome, allowing the distinction of wildtype and kanamycin resistant strains. Further, kanamycin also alters the mVOC metabolome in both the wildtype and *kan*^*R*^ strain; as seen in Fig. 5D, the mVOCs extracted from wildtype *Y. pestis* is differentiated in media with and without kanamycin (this phenomenon is also observed with the *Y. pestis kan*^*R*^ strain). It’s noteworthy that this is directly in line with our above observations of environmental effectors impacting the mVOC metabolome (see the “Influence of environmental effectors on the derived mVOC profile” section).

While the scree plots from the PCAs indicate that multiple metabolites contribute to the differentiation of wildtype and kanamycin resistant strains (data not shown), to pinpoint key differing metabolites we performed a univariate comparison of the 15 min mVOC metabolomes and identified several features with statistically significant change (Table 2). Specifically, when the mVOC metabolome derived from the wildtype strain of *Y. pestis* (cultured in media without kanamycin) is compared to the mVOC metabolome derived from the *kan*^*R*^ strain (cultured in media with kanamycin), relative to the wildtype strain the kanamycin resistant strain generates a ~ 900 fold and ~ 50,000 fold increase in the polyamines ethylenediamine and dimethyl urea, respectively (Table 2). Intriguingly, polyamines have been shown to enhance the catalytic activity of kinases^25^, as well as promote biofilm formation in *Y. pestis*^26^. In contrast, in a parallel analysis comparing *F. tularensis* wildtype to the kanamycin resistant strain, neither of these polyamines were found elevated in the *kan*^*R*^ strain (Table 2). It’s also notable that *F. tularensis* does not generally form biofilms^27^.

**Table 2:** Metabolic features associated with kanamycin resistance in *Y. pestis* or *F. tularensis*.

	log_2_Fold Change^b^		AMDIS score
	(Resistant/Wildtype)	p-value	
***Y. pestis associated features***^**a**^			
Ethylenediamine	9.77	6.6 × 10^–7^	93
Urea, N,N-dimethyl-	15.57	2.1 × 10^–10^	92
Isopropylhydroxylamine	− 13.03	7.1 × 10^–9^	95
3-(2-chloro-5-hydroxyphenyl)-2-methylquinazolin-4-one	− 9.59	8.4 × 10^–7^	91
***F. tularensis associated features***			
2,3-Dimethylhexane	− 12.95	7.9 × 10^–9^	90
Methyl-alpha-aminobutyrate	− 11.71	4.4 × 10^–8^	90
2-Nitroethyl propionate	− 17.13	2.4 × 10^–11^	90

^a^Criteria for inclusion: |log_2_(Fold Change)|≥ 9.50 and p-value < 0.05.

^b^Positive values denote the elevation of the metabolite in the resistant cohort when compared to the wildtype. Negative values reflect the suppression of the metabolite in the resistant cohort with respect to the wildtype cohort.

Differential comparison also revealed that kanamycin resistant *Y. pestis* produces significantly lower amounts of 3-(2-chloro-5-hydroxyphenyl)-2-methylquinazolin-4-one and isopropylhydroxylamine, relative to the wildtype strain (~ 800 and 8,000 fold lower, respectively). The quinazolinone alkaloids are a large family of secondary metabolites produced by plants, fungi, and bacteria^28–30^. Alterations to primary metabolism is well known to alter production of secondary metabolites^31^. Hence, the metabolic shift associated with *kan*^*R*^ may lead to reduced quinazolinone production in the resistant strain. Similarly, decreased isopropylhydroxylamine in the *kan*^*R*^ strain may also reflect an overall metabolic shift in the bacteria. *Y. pestis* CO92 is nitrate positive^32^ and bacterial hydroxylamines are associated with bacterial nitrate and nitrite reduction^33^, a process requiring an iron atom^34^. Intriguingly, the Yersinia iron-chelating siderophore yersiniachelin is synthesized from hydroxylamines^35^. Hence subtle perturbation of this hydroxylamine cycle is expected to largely affect cellular hydroxylamine levels.

Table 2 also lists molecular features found statistically depleted in the kanamycin resistant strain of *F. tularensis*, relative to the corresponding wildtype strain. These too are likely the consequence of multifactor alterations to bacterial metabolism. Overall, the mVOC profiling indicates that a 6 fiber simulti-hSPME analysis can differentiate wildtype and kanamycin resistant strains of *Y. pestis* and *F. tularensis*. Relative to a 6 fiber multi-hSPME-based analysis, simulti-hSPME reduces the analysis time ~ 5 to 6 fold. It’s noteworthy that optimization of the 30 min chromatographic run could shorten the analysis time even further.

### Mouse VOC analysis: differentation of healthy and bacteria-infected animals

Given that mVOCs emanating from in vitro cultures differentiate the bacterial strains, we next sought to assess our ability to differentiate bacteria-infected mice from uninfected mice, based upon their associated VOC profiles. As detailed in Supplementary Figures S1–S6, we tried three different simulti-hSPME-based approaches to this analysis; (1) collecting the mouse VOCs in an airtight gas sampling bag, then connecting the bag to our extraction device and extracting the contents via simulti-hSPME, (2) placing the mice in a customized sampling chamber configured with ports for simulti-hSPME, and (3) fabricating a simulti-hSPME-based remote sensor and placing it into the mouse housing cage. Of these three approaches, only the latter produced useful chromatograms (numerous peaks in ample abundance), but only with an extended extraction duration. Since we wanted a more rapid analysis, we sought an alternative approach to sampling the VOCs.

**Figure 6:**
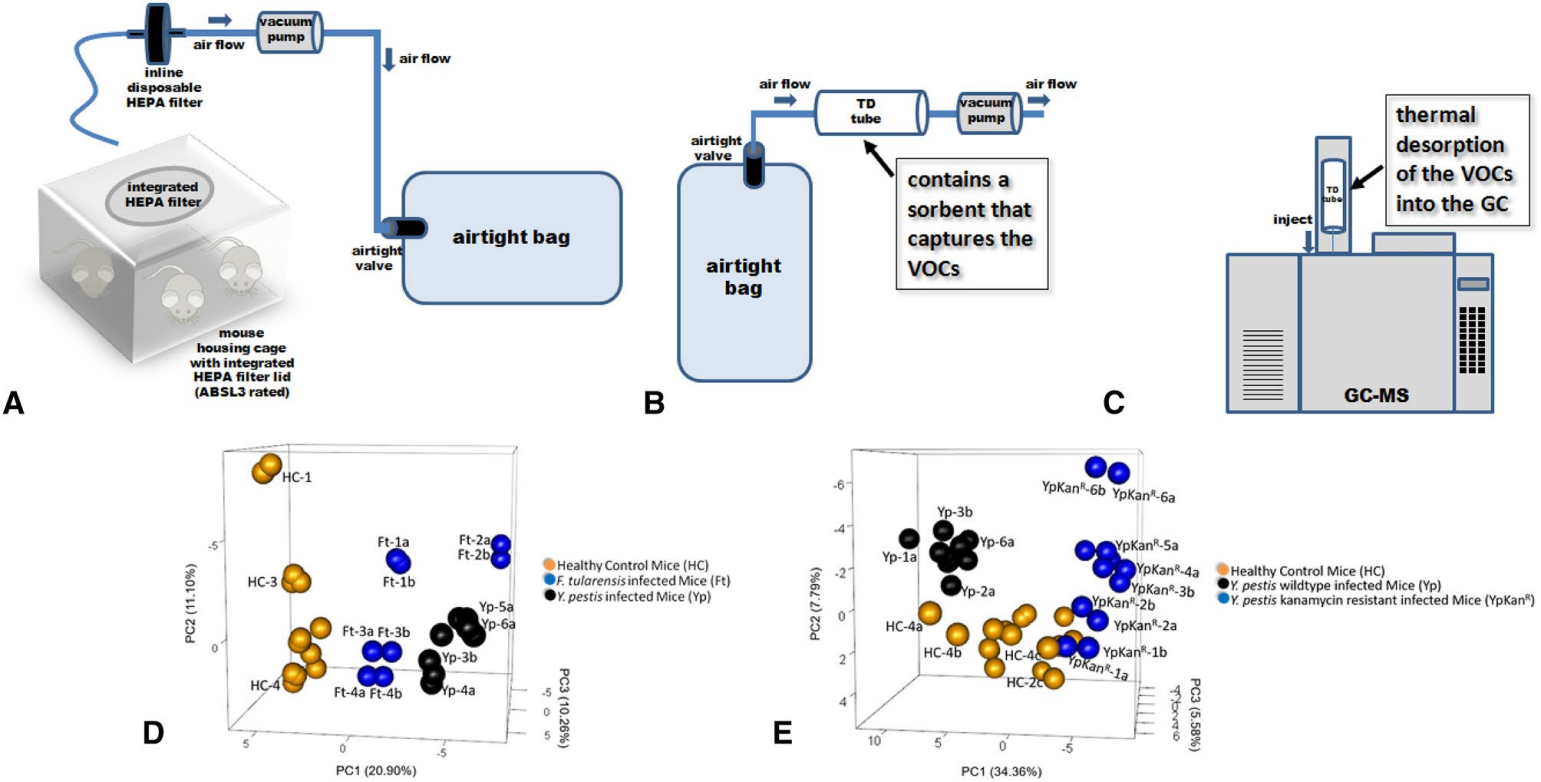
Differentiation of Healthy and Infected Mice using TD-GC–MS. (**A**) The air from within a mouse housing cage is captured in a 10 L airtight ALTEF bag using a vacuum pump (operated at a flow rate of 20 L/min). (**B**) The content of the airtight bag is then vacuumed through a thermal desorption tube, which contains a sorbent (Tenax TA) that captures the VOCs. (**C**) The thermal desorption tube is subsequently placed into an appropriately equipped GC, where the captured VOCs are thermally desorbed directly into the GC–MS for analysis. Our TD–GC–MS system has a cryotrap connected to liquid nitrogen at the head of the GC column, which captures and concentrates the VOCs thermally desorbed from the TD tube. When the cryotrap is subsequently heated, the analytes are released into the GC column for analysis. (**D**) TD–GC–MS was used to analyze the mouse-associated VOC metabolomes in three separate cohorts of mice; (1) uninfected healthy control mice (orange spheres), (2) wildtype *F. tularensis* SCHU S4 infected mice (blue spheres), and (3) wildtype *Y. pestis* CO92 infected mice (black spheres). The PCA plot of the resulting mouse VOC metabolomes is shown, with each sphere representing the VOC metabolome derived from the indicated mouse cohort on the indicated day. Each cohort contained 5 mice, the infection rate was 100%, and the entire study was performed in duplicate (on separate occasions; replicates are designated with the letters a and b in the plot). The VOCs were collected from the mouse cages daily, starting from day 1 post exposure to the clinical endpoint (labeled accordingly in the plot). Visible signs of infection were noted at day 1 (*F. tularensis*) and day 3 (*Y. pestis*). The VOC metabolomes clearly permit differentiation of healthy mice from those that are infected, and enable diagnosis/differentiation of infection with *F. tularensis* versus *Y. pestis*. (**E**) TD–GC–MS was used to analyze the mouse-associated VOC metabolomes in three separate cohorts of mice; (1) uninfected healthy control mice (orange spheres), (2) wildtype *Y. pestis* CO92 infected mice (black spheres), and (3) kanamycin resistant *Y. pestis* CO92 infected mice (blue spheres). A PCA plot of the resulting mouse VOC metabolomes is shown, with each sphere representing the VOC metabolome derived from the indicated mouse cohort on the indicated day. Each cohort contained 5 mice, the infection rate was 100%, and the entire study was performed in duplicate (on separate occasions). The VOCs were collected from the mouse cages daily, starting from day 1 to day 6 post exposure. Visible signs of infection were noted at day 3 post exposure in both infection types. The VOC metabolomes clearly permit differentiation of healthy mice from those that are infected, and enable diagnosis/differentiation of infections resulting from either an antimicrobial resistant or sensitive strain.

Unlike hSPME which involves passive sampling, thermal desorption (TD) uses a vacuum pump to actively pull the VOCs through a tube containing adsorbent material (see Fig. 6A–C). As such, a large volume of air is more rapidly sampled, and the VOCs are concentrated on the sorbent prior to analysis^36^. As with hSPME, the sorbent chemistry in the TD tube dictates the nature of the analytes isolated from a sample.

To perform the TD-based mouse VOC analysis, 15 female BALB/c mice were equally divided into three cohorts that underwent whole body exposure to either aerosolized sterile liquid culture media (the healthy control cohort), an aerosolized liquid culture of wild type *F. tularensis* SCHU S4, or an aerosolized liquid culture of wildtype *Y. pestis* CO92. The infection rate was 100% in the *F. tularensis* and *Y. pestis* exposure cohorts (i.e. all 5 of 5 mice in each cohort were infected with agent; the mice were exposed at ~ 100 × LD_50_ for each pathogen). Beginning day 1 and continuing daily to the clinical endpoint (day 4 post exposure to *F. tularensis*; day 6 post exposure to the *Y. pestis* strains), the mouse VOCs were collected from each cohort’s housing cage, as illustrated in Fig. 6A (each cage housed all 5 members of the cohort). Immediately following collection, VOC analysis was performed by TD-GC–MS, as depicted in Fig. 6B, C. The time to complete steps 6A through 6C is ~ 1 h per cohort. Informatic analysis of the resulting GC–MS chromatograms, including comparison to a mass spectral database, produced a complete VOC metabolome for each of the three cohorts, at each time of the analysis (day 1–4 with *F. tularensis*; day 1–6 with the *Y. pestis* strains). This entire experiment was then repeated, on a separate occasion, to generate duplicate VOC metabolomes for each of the three cohorts, at each time point of analysis. Replicate data was then compared and only the VOCs appearing in both corresponding replicates were included in the subsequent analysis. A principal component analysis was then performed using the derived VOC metabolomes. As seen in the resulting PCA plot (Fig. 6D), the mouse-derived VOC fingerprints clearly differentiate the healthy mice from those that are infected. Furthermore, the graphical distinction of mice infected with *F. tularensis* from those infected with *Y. pestis* indicates how the mouse-derived VOC metabolomes could potentially be used for diagnosis of infection, and hints to the promise of the approach as a rapid method for clinical diagnostics.

Finally, an identical approach was used to compare mouse-derived VOC metabolomes obtained from cohorts of healthy control mice, wildtype *Y. pestis* infected mice, and kanamycin resistant *Y. pestis* infected mice. As seen in Fig. 6E, the VOCs differentiate the infected mice from the healthy controls, and further, mice infected with the engineered antibiotic resistant strain from mice infected with the wildtype strain of *Y. pestis*.

## Conclusions

By utilizing a 6-fiber multi-hSPME-based approach, uniquely identifying mVOC fingerprints were derived for liquid cultures of *F. tularensis spp. novicida*, *F. tularensis spp. tularensis, Y. pestis* A1122, *Y. pestis* CO92, *B. cenocepacia*, and *B. neotomae*. As anticipated, given that phenomenon such as nutrient depletion dependent gene expression, carbon catabolite repression of gene expression, and quorum sensing are all well known to influence bacterial metabolism, it is no surprise that alterations in media composition (specifically herein, a metal salt) alters the resulting mVOC fingerprint. However, comparing the mVOC profiles from bacteria cultured in various media types, it’s possible to define a core metabolome comprising mVOCs consistently produced in all of the media types tested. This so called core metabolome can be used to uniquely identify each of the bacteria tested, regardless of the media composition. Hence, while a database of mVOC profiles could be created for select environmental conditions of particular interest (for example open wounds, surgical procedures, urban environments, etc.), it appears possible that a core mVOC profile could be defined for each bacteria that will permit microbial identification in a wide array of environmental conditions. Additional work is needed to evaluate this.

In the course of this investigation we were also able to differentiate wildtype and kanamycin resistant strains of both *Y. pestis* and *F. tularensis*. Future work will assess if microbes harboring other forms of antimicrobial resistance can also be discerned by mVOC fingerprints, as the diagnostic potential in medicine and biodefense is obvious. And by designing and developing the simulti-hSPME device, we are able to perform the 6-fiber extractions simultaneously, thereby dramatically reducing our overall analysis time (~ 2 h to complete in triplicate). Optimization of the chromatography conditions will no doubt reduce this analysis time even further.

While highly effective for in vitro analyses, in our hands simulti-hSPME does not lend itself for use in the rapid diagnosis of mice infected with *F. tularensis* or *Y. pestis*, although it does show potential for use as a remote sensor, with prolonged extraction duration. On the other hand, by rapidly sampling large volumes of air in a short period of time, TD coupled with GC–MS enabled us to use mouse-derived VOCs to differentiate healthy mice from those infected with *F. tularensis* or *Y. pestis*, and even differentiate infection with wildtype *Y. pestis* from infection with a kanamycin resistant strain. Hence, TD–GC–MS shows great potential for use in rapid clinical diagnostics, with clear civilian and military application to address the threat of future epidemics/pandemics and/or possible terrorist use of pathogenic organisms.

## Supplementary information

Supplementary Information.

## Data Availability

The datasets generated during and/or analyzed during the study are available from the corresponding author on request.

## References

[1] Lazcka O, Del Campo FJ, Muñoz FX (2007). Pathogen detection: a perspective of traditional methods and biosensors. Biosens. Bioelectron..

[2] Lasch P (2015). Identification of highly pathogenic microorganisms by matrix-assisted laser desorption ionization-time of flight mass spectrometry: results of an interlaboratory ring trial. J. Clin. Microbiol..

[3] Singhal N, Kumar M, Kanaujia PK, Virdi JS (2015). MALDI-TOF mass spectrometry: an emerging technology for microbial identification and diagnosis. Front. Microbiol..

[4] Couch RD (2015). Alcohol induced alterations to the human fecal VOC metabolome. PLoS ONE.

[5] Couch RD (2013). The approach to sample acquisition and its impact on the derived human fecal microbiome and VOC metabolome. PLoS ONE.

[6] Li RW (2012). Alterations in the porcine colon microbiota induced by the gastrointestinal nematode *Trichuris suis*. Infect. Immun..

[7] Dixon E (2011). Solid-phase microextraction and the human fecal VOC metabolome. PLoS ONE.

[8] Bos LDJ, Sterk PJ, Schultz MJ (2013). Volatile metabolites of pathogens: a systematic review. PLoS Pathog..

[9] Tait E, Perry JD, Stanforth SP, Dean JR (2014). Identification of volatile organic compounds produced by bacteria using HS-SPME-GC–MS. J. Chromatogr. Sci..

[10] Zhu J, Bean HD, Kuo Y-M, Hill JE (2010). Fast detection of volatile organic compounds from bacterial cultures by secondary electrospray ionization-mass spectrometry. J. Clin. Microbiol..

[11] Lim SH (2014). Colorimetric sensor array allows fast detection and simultaneous identification of sepsis-causing bacteria in spiked blood culture. J. Clin. Microbiol..

[12] Mellors TR, Rees CA, Wieland-Alter WF, von Reyn CF, Hill JE (2017). The volatile molecule signature of four mycobacteria species. J. Breath Res..

[13] Rees CA (2018). Detection of high-risk carbapenem-resistant *Klebsiella pneumoniae* and *Enterobacter cloacae* isolates using volatile molecular profiles. Sci. Rep..

[14] Conchas RF, Carniel E (1990). A highly efficient electroporation system for transformation of *Yersinia*. Gene.

[15] Maier TM (2004). Construction and characterization of a highly efficient *Francisella* shuttle plasmid. Appl. Environ. Microbiol..

[16] Arthur CL, Pawliszyn J (1990). Solid phase microextraction with thermal desorption using fused silica optical fibers. Anal. Chem..

[17] Pawliszyn J (2000). Theory of solid phase microextraction. J. Chromatogr. Sci..

[18] Agar SL (2008). Characterization of a mouse model of plague after aerosolization of *Yersinia pestis* CO92. Microbiol. Read. Engl..

[19] Anderson NW (2012). Effects of solid-medium type on routine identification of bacterial isolates by use of matrix-assisted laser desorption ionization-time of flight mass spectrometry. J. Clin. Microbiol..

[20] Wieme AD (2014). Effects of growth medium on matrix-assisted laser desorption-ionization time of flight mass spectra: a case study of acetic acid bacteria. Appl. Environ. Microbiol..

[21] Chudobova D (2015). The effect of metal ions on *Staphylococcus aureus* revealed by biochemical and mass spectrometric analyses. Microbiol. Res..

[22] Blundell MR, Wild DG (1969). Inhibition of bacterial growth by metal salts. A survey of effects on the synthesis of ribonucleic acid and protein. Biochem. J..

[23] Integrative analysis of fitness and metabolic effects of plasmids in Pseudomonas aeruginosa PAO1. *ISME J*. https://www.nature.com/articles/s41396-018-0224-8. 10.1038/s41396-018-0224-8 PMC6246594 30097663

[24] Zampieri M, Zimmermann M, Claassen M, Sauer U (2017). Nontargeted metabolomics reveals the multilevel response to antibiotic perturbations. Cell Rep..

[25] Meksuriyen D (1998). Formation of a complex containing ATP, Mg2+, and spermine structural evidence and biological significance. J. Biol. Chem..

[26] Patel CN (2006). Polyamines are essential for the formation of plague biofilm. J. Bacteriol..

[27] Champion AE, Catanzaro KCF, Bandara AB, Inzana TJ (2019). Formation of the *Francisella tularensis* biofilm is affected by cell surface glycosylation, growth medium, and a glucan exopolysaccharide. Sci. Rep..

[28] Kshirsagar UA (2015). Recent developments in the chemistry of quinazolinone alkaloids. Org. Biomol. Chem..

[29] Shang X-F (2018). Biologically active quinoline and quinazoline alkaloids part I. Med. Res. Rev..

[30] Shang X-F (2018). Biologically active quinoline and quinazoline alkaloids part II. Med. Res. Rev..

[31] Drew SW, Demain AL (1977). Effect of primary metabolites on secondary metabolism. Annu. Rev. Microbiol..

[32] Zhou D (2004). Genetics of metabolic variations between *Yersinia pestis* biovars and the proposal of a new biovar, microtus. J. Bacteriol..

[33] Lindsey GA, Rhines CM (1932). The production of hydroxylamine by the reduction of nitrates and nitrites by various pure cultures of bacteria 1. J. Bacteriol..

[34] González PJ, Correia C, Moura I, Brondino CD, Moura JJG (2006). Bacterial nitrate reductases: molecular and biological aspects of nitrate reduction. J. Inorg. Biochem..

[35] Rakin A, Schneider L, Podladchikova O (2012). Hunger for iron: the alternative siderophore iron scavenging systems in highly virulent *Yersinia*. Front. Cell. Infect. Microbiol..

[36] Woolfenden, E. Thermal desorption for gas chromatography. in* Gas Chromatography* (Elsevier, 2012).

