# Supplementary material for: VOC fingerprints: metabolomic signatures of biothreat agents with and without antibiotic resistance
Source: Sci Rep. 2020 Jul 16;10:11746. doi: 10.1038/s41598-020-68622-x (PMC7367350; doi:10.1038/s41598-020-68622-x)
Supplement: Supplementary file 1 — Supplementary Information. [file 41598_2020_68622_MOESM1_ESM.pdf]

VOC Fingerprints: Metabolomic Signatures of Biothreat Agents With and Without Antibiotic Resistance

Allyson Dailey[1], Jessica Saha[1], Fatima Zaidi[1], Hafsa Abdirahman[1], Amanda Haymond[1], Farhang Alem[2], Ramin Hakami[2], and Robin Couch[1*]

[1]Department of Chemistry and Biochemistry, George Mason University, Manassas, Virginia, United States of America
[2]National Center for Biodefense and Infectious Diseases, School of Systems Biology, George Mason University, Manassas, Virginia, United States of America

*Corresponding author

Email: rcouch@gmu.edu

Supplementary Information:

Three simulti-hSPME GC-MS-based approaches to mouse VOC analysis.

1. Chamber + ALTEF Bag. In this approach, we configured a commercially purchased airtight mouse chamber to interface directly with an ALTEF bag (Supplementary Figure S1). In this arrangement, the mice are placed in the chamber and are free to move around at will. HEPA filtered air is applied to the chamber (air inflow), and the outflow air is collected into an ALTEF bag. We then connect the ALTEF bag to our extraction device and extract the contents via simulti-hSPME, using our choice of extraction duration.

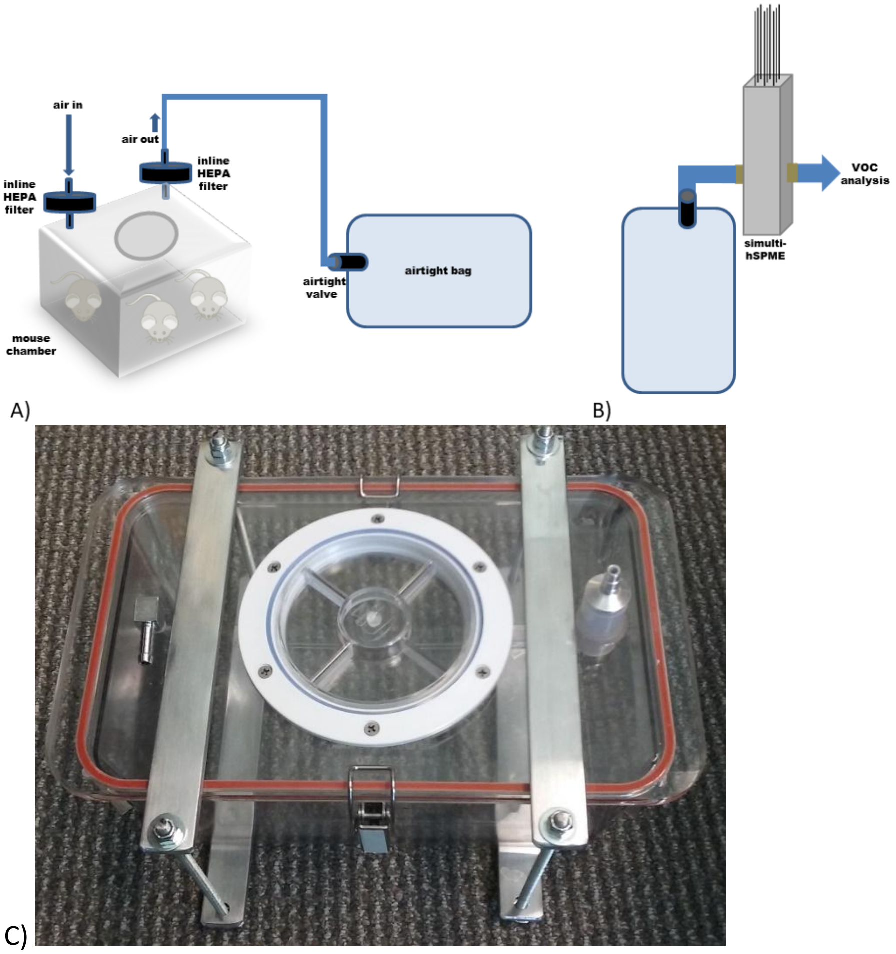

Supplementary Figure S1. The chamber + ALTEF bag approach to analyzing VOCs emanating from the mice. A) A cohort of mice (uninfected or infected) is placed in the airtight mouse chamber. Compressed air travels into the chamber through a HEPA filter forcing air from the chamber out and into an airtight ALTEF bag. B) The captured air is subsequently analyzed by simulti-hSPME coupled with GC-MS. C) The commercially purchased airtight chamber is shown, with a custom fabricated bracket system to clamp down the lid, thereby tightly sealing the chamber.

As seen in Supplementary Figure S2, using a 15 min extraction duration, the resulting chromatogram of healthy mouse-associated VOCs is relatively sparse, with most peaks being of low intensity (this is indicative of low analyte abundance in the collected air sample). However,

prolonged extraction (24 hr in Supplementary Figure S2) greatly improves the resulting signal intensity and increases the total number of peaks present in the chromatogram, indicating that optimization of extraction duration (between 15 min and 24 hr) will enable this approach for effective mouse VOC analysis. While this approach shows promise, our preference is for a more rapid diagnostic, so we next evaluated a simulti-hSPME-based analysis directly from the mouse chamber (described below).

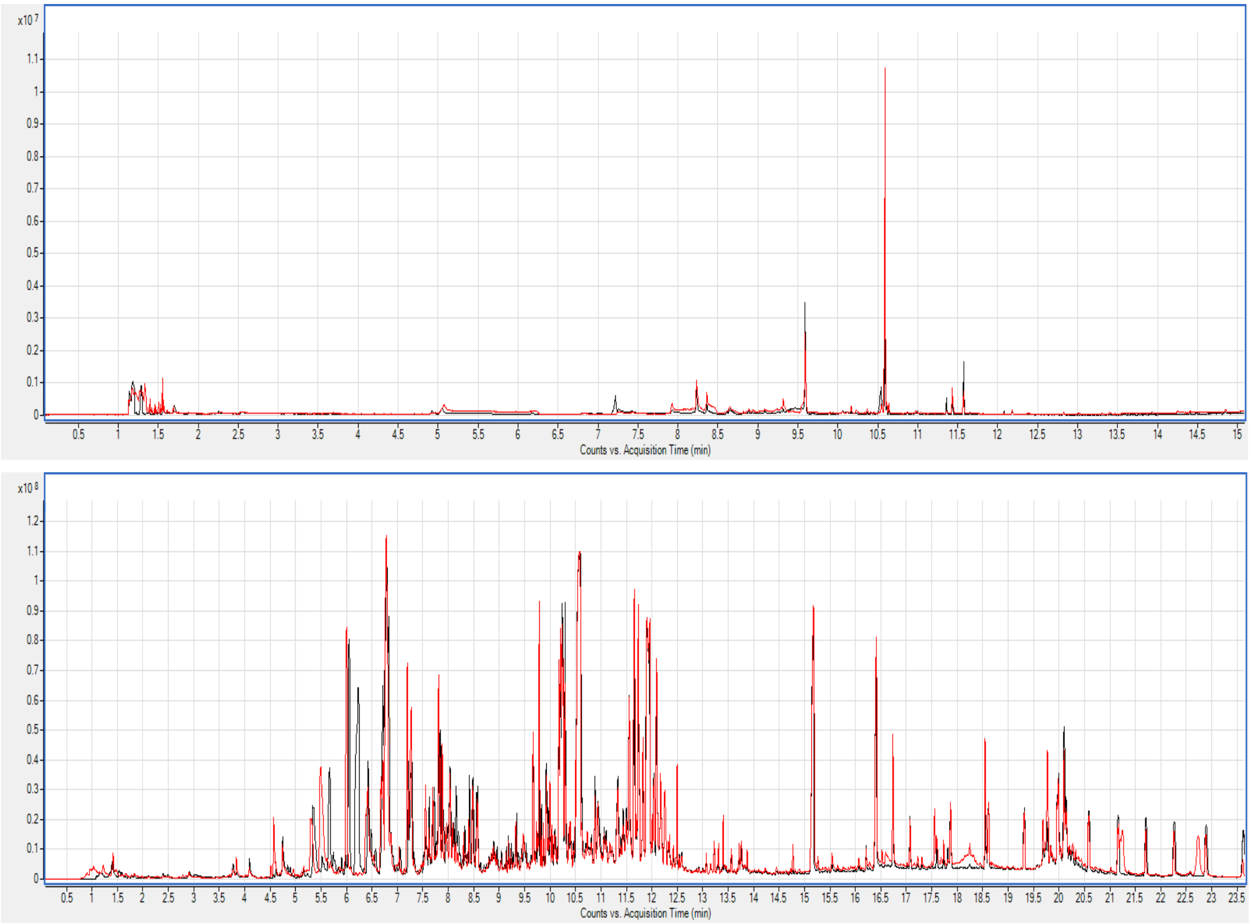

Supplementary Figure S2. The resulting chromatograms from the chamber + ALTEF bag approach to analyzing mouse VOCs. A comparison of mouse associated VOCs (colored red) to the chamber blank (colored black) is shown. A 6-fiber analysis was performed, using a 15 min extraction duration (top overlay) or a 24 hr extraction duration (bottom overlay). As expected, greater signal intensity and complexity is achieved with the longer hSPME extraction duration. Fibers used were PEG 60 µm, PDMS/DVB 65 µm, PDMS 100 µm, DVB/CAR/PDMS 50/30 µm, PA 85 µm, and CAR/PDMS 85 µm. This analysis was performed on the GC-MS.

2. Chamber + Direct Simulti-hSPME. In this approach to mouse VOC analysis, we modified the mouse chamber to contain six ports with septum to permit the direct simulti-hSPME-based analysis of the air within the chamber (Supplementary Figure S3). As shown in Supplementary Figure S4, a GC-MS-based 6-fiber simulti-hSPME analysis with 1 hr extraction duration yielded

a VOC chromatogram with relatively few peaks, most having low signal intensity. Further, the mouse VOC fingerprint is difficult to distinguish from one derived from an empty chamber, indicative of low abundance mouse-associated VOCs within the mouse chamber when the mice are present. Increased extraction durations and alternative fiber chemistry types were each evaluated with this approach, with only the former significantly improving the resulting chromatograms (data not shown). In light of this, rather than place the mice in the mouse chamber for extended extraction durations, we instead developed a ‘remote sensor’ which we placed in the housing mouse cage where the cohort of mice spend most of their time eating, sleeping, and residing (as detailed in the next section).

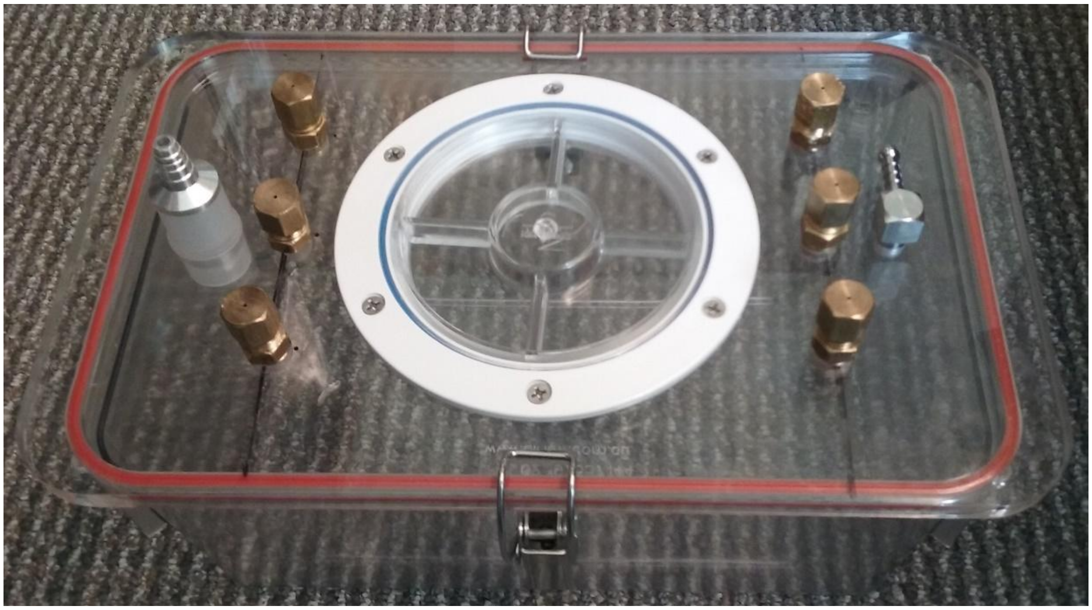

Supplementary Figure S3. The chamber + direct simulti-hSPME approach to analyzing VOCs emanating from the mice. In this approach, a cohort of mice (uninfected or infected) is placed in the mouse chamber. HEPA filters passively allow air to flow in and out of the cage. Using the six ports we integrated into the chamber lid, the air within the cage is analyzed by simulti-hSPME coupled with GC-MS.

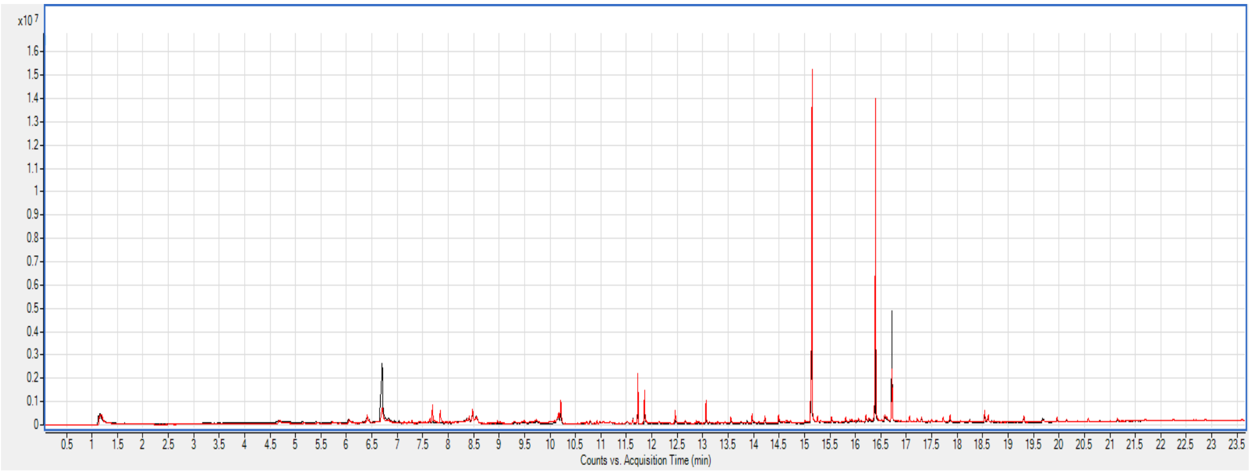

Supplementary Figure S4. The resulting chromatograms from the chamber + direct simulti-hSPME approach to analyzing mouse VOCs. A comparison of mouse associated VOCs (colored red) to the chamber blank (colored black) is shown. A 6-fiber analysis was performed, using a 1 hr extraction duration. Fibers used were PEG 60 µm, PDMS/DVB 65 µm, PDMS 100 µm, DVB/CAR/PDMS 50/30 µm, PA 85 µm, and CAR/PDMS 85 µm. The analysis was performed using the GC-MS. The presence of few peaks with low signal intensity is indicative of low abundant VOCs, which prompted us to evaluate an alternative approach to VOC analysis.

3. Remote Sensor. As indicated above, we found that extended duration simulti-hSPME of the air captured within an ALTEF bag or of the air within an airtight mouse chamber produced more desirable chromatograms than did short extraction durations. Hence, rather than transfer the cohort of mice to the mouse chamber for analysis, we instead fabricated a remote sensor to detect mouse-associated VOCs directly in the cages used to house the mice (Supplementary Figure S5). As seen in Supplementary Figure S6, while a 24 hr extraction duration produces numerous peaks in the resulting chromatogram, it is difficult to differentiate the mouse-associated VOCs from an empty cage. On the other hand, a 72 hr extraction duration enhances the chromatogram considerably, clearly differentiating the mouse-associated VOCs from the empty cage. While this approach demonstrates potential for use as a remote sensor, for our goal we strive to keep the analysis time as short as possible. Further analysis is required to determine the effectiveness of extraction durations between 24 and 72 hrs.

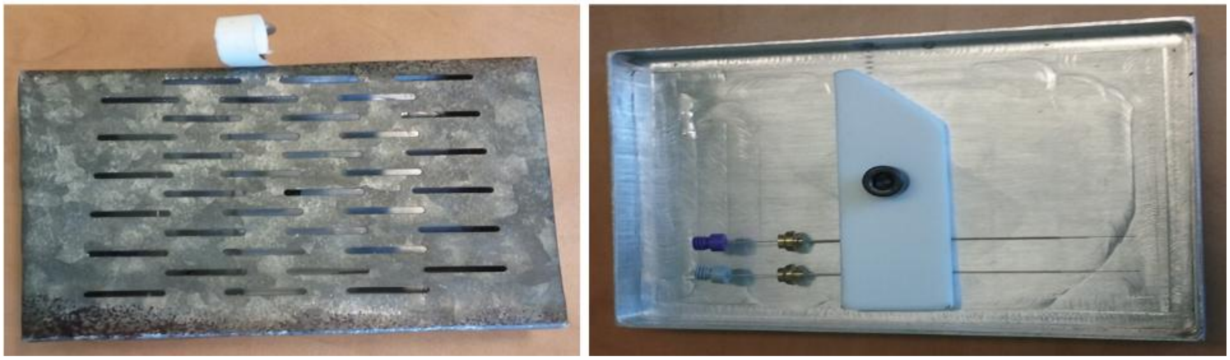

Supplementary Figure S5. A simulti-hSPME-based remote sensor of VOCs. The sensor is approximately 15x10 cm in size and has a perforated lid (left photo) that allows air to freely pass to the inside (right photo). SPME fibers are secured to the inside of the sensor, the lid is attached, and the sensor is placed in the cage with the cohort of mice. After the desired extraction duration, the SPME fibers are removed from the sensor and analyzed by GC-MS.

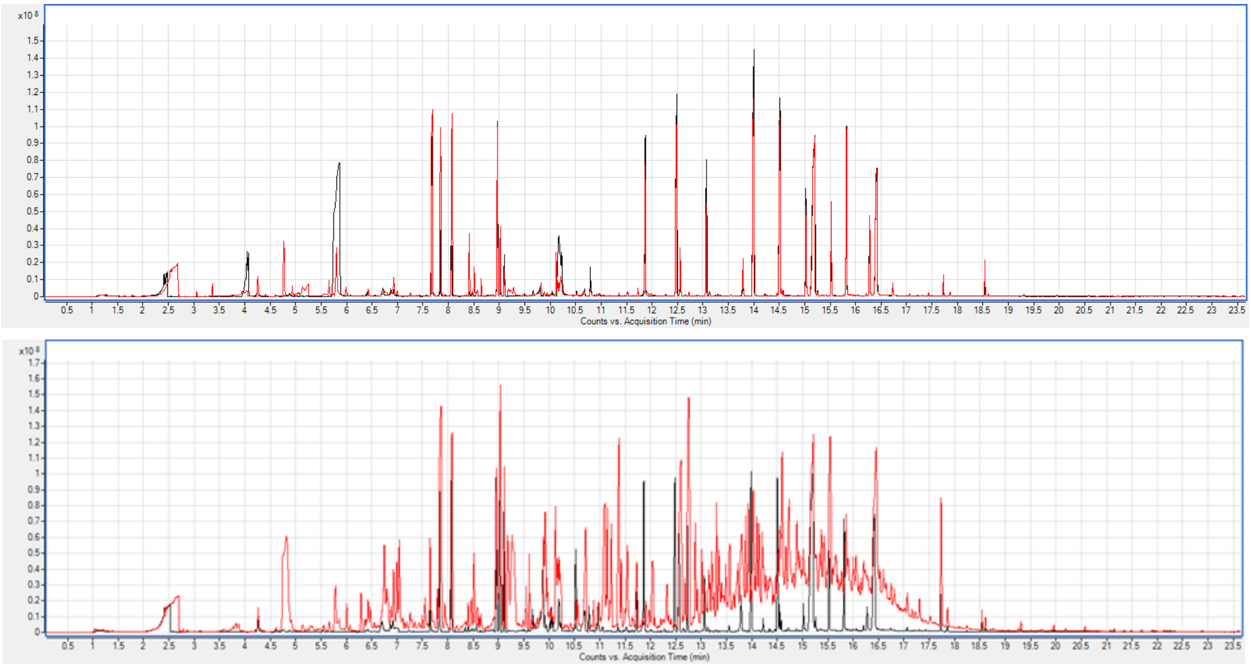

Supplementary Figure S6. Chromatograms derived from the remote sensor approach to analyzing mouse VOCs. In the top chromatographic overlay, the extracted mouse-associated VOCs (colored red) are compared to the cage blank (colored black), obtained with a 24 hr extraction duration. Only subtle differences distinguish these chromatograms from each other. In the bottom overlay, mouse-associated VOCs (colored red) are compared to the cage blank (colored black), obtained with a 72 hr extraction duration. Due to the increased number of mouse-associated analytes, this extended extraction duration clearly differentiates the conditions. All analyses were performed with the GC-MS using PEG 60 µm, PDMS/DVB 65 µm, PDMS 100 µm, DVB/CAR/PDMS 50/30 µm, PA 85 µm, and CAR/PDMS 85 µm fibers.
